# Predictors of all-cause mortality among 514,866 participants from the Korean National Health Screening Cohort

**DOI:** 10.1371/journal.pone.0185458

**Published:** 2017-09-28

**Authors:** Choonghyun Ahn, Yunji Hwang, Sue K. Park

**Affiliations:** 1 Department of Preventive Medicine, Seoul National University College of Medicine, Seoul, Korea; 2 Department of Biomedical Science, Seoul National University Graduate School, Seoul, Korea; 3 Cancer Research Institute, Seoul National University College of Medicine, Seoul, Korea; Istituto Di Ricerche Farmacologiche Mario Negri, ITALY

## Abstract

**Background:**

There is not enough evidence regarding how information obtained from general health check-ups can predict individual mortality based on long-term follow-ups and large sample sizes. This study evaluated the applicability of various health information and measurements, consisting of self-reported data, anthropometric measurements and laboratory test results, in predicting individual mortality.

**Methods:**

The National Health Screening Cohort included 514,866 participants (aged 40–79 years) who were randomly selected from the overall database of the national health screening program in 2002–2003. Death was determined from causes of death statistics provided by Statistics Korea. We assessed variables that were collected at baseline and repeatedly measured for two consecutive years using traditional and time-variant Cox proportional hazards models in addition to random forest and boosting algorithms to identify predictors of 10-year all-cause mortality. Participants’ age at enrollment, lifestyle factors, anthropometric measurements and laboratory test results were included in the prediction models. We used c-statistics to assess the discriminatory ability of the models, their external validity and the ratio of expected to observed numbers to evaluate model calibration. Eligibility of Medicaid and household income levels were used as inequality indexes.

**Results:**

After the follow-up by 2013, 38,031 deaths were identified. The risk score based on the selected health information and measurements achieved a higher discriminatory ability for mortality prediction (c-statistics = 0.832, 0.841, 0.893, and 0.712 for Cox model, time-variant Cox model, random forest and boosting, respectively) than that of the previous studies. The results were externally validated using the community-based cohort data (c-statistics = 0.814).

**Conclusions:**

Individuals’ health information and measurements based on health screening can provide early indicators of their 10-year death risk, which can be useful for health monitoring and related policy decisions.

## Introduction

General health check-ups are a screening procedure targeting the currently healthy population to detect diseases earlier and to intervene to better prevent chronic diseases. Check-ups usually include a medical history, anthropometric measurements and laboratory tests such as simple blood and urine tests. These visits might help detect and prevent chronic diseases, but there is insufficient evidence regarding the effectiveness of interventions based on periodic health check-ups and the predictive value of the information obtained. Two prior systematic reviews of clinical trials of general health check-ups were critical of the outcomes, with general check-ups not reducing all-cause, cancer or cardiovascular disease mortality [1, 2]. On the other hand, two nationwide population-based cohort studies in Korea and Taiwan reported a favorable effect of health check-ups, such as lower all-cause and cardiovascular disease (CVD) mortality rate and early treatment of hypertension, diabetes, and dyslipidemia [3, 4]. The study populations and areas included in the systematic reviews were limited, as they included studies on European descendants in developed countries and the average cost of general health check-ups (£423 (near $464) for Eurohealth in 2009 [5]) seemed to be higher than that of the national health insurance coverage in the latter cohort studies. The Korea National Health Insurance Service (NHIS) provides a mandatory biennial general health check-up for people aged 40 years and over that does not require copayment and reimburses approximately $40 to medical providers upon return of individuals’ health check-up and report [6, 7]. The NHIS also provides health check-ups for blue-collar workers every year. It covers the entire employed and unemployed population over the age of 40 years. 74.8% of eligible population participated in the biennial health check-up in 2014[8].

The difference in results between the favorable effect of general health check-ups in Korea and Taiwan and the lack of beneficial effect in the reviews of European descendants may be due to differences in whether the general health check-up program was covered by mandatory policies under cheap or no copayment. The results of ‘natural experiments’ in entire populations provide compelling evidence [9]. Previous studies have assessed the predictive values of risk factors and developed all-cause mortality predictors with self-reported health status in the UK and US [10, 11], but they did not develop a mortality predictor using a combination of self-reported health status and objective test results to represent the mortality risk of the general population.

Although having periodic health check-ups cannot be mortality predictor by itself, the collected information from heath check-ups can be useful to predict of mortality risk and to apply personalized prevention and intervention strategies. This study examined the applicability of information from self-reported questionnaires, anthropometric measurements and laboratory test results collected from the general health check-up program as an effective predictor of mortality among the healthy or asymptomatic population based on the large-scale nationwide database in Korea.

## Materials and methods

### Data collection

The NHIS database includes various health check-up items based on physicians’ counseling and physical examinations, dentists’ dental examinations, and health examinees’ questionnaire results and anthropometric measurements. In addition, systolic and diastolic blood pressure (SBP, DBP), vision, hearing, and chest x-ray imaging results were collected. Blood and urine samples were collected, and laboratory tests were performed, including dipstick urine tests (occult blood, glucose and protein), complete blood count (CBC), fasting blood glucose (FBG), and serum levels of aspartate aminotransferase/alanine transaminase (AST/ALT), gamma-glutamyl transpeptidase (ϒ-GTP), total cholesterol, high-density lipoprotein (HDL)-cholesterol, triglycerides (TG) and creatinine.

Among the participants aged 40–79 years who participated in the biennial national health screening program covered in the Korea National Health Insurance cooperation in 2002 and 2003, 10% of all participants were randomly selected to form the National Health Insurance Service-National Health Screening Cohort (NHIS-HEALS). As a result, 514 866 subjects were selected to construct the cohort. Between 2002 and 2008, each subject had participated in the national health screening program 1–7 times. 67 737 participants undertook once, on the other hand 38 222 participants undertook 7 times of health check-ups. The data included information from the repeated health check-ups. The date and cause of death was identified from the records of Statistics Korea.

The health check-up collects survey data, body measurements and blood and urine test results. Between 2002 and 2008, the questionnaire included past medical history and family history (liver disease, stroke, cancer, heart disease, diabetes mellitus, or hypertension, drinking frequency and amount, and smoking frequency and amount. In the same period, anthropometric measurements (weight and height), BP and laboratory testing results (fasting blood glucose (FBG), total cholesterol level, ALT, AST, GGT, hemoglobin, urine pH, urine occult blood, and urine protein). In total, 546 subjects whose body mass indexes (BMIs) were missing were excluded.

Based on a modified version of the guidelines of the American Diabetes Association (ADA) in 2016 [12], study subjects were classified by measured FBG levels into 5 groups: ‘Hyperglycemic crisis’ (≥200 mg/dL, 11.1 mmol/L); ‘Diabetes’ (126–199 mg/dL, 7.0–11.0 mmol/L); ‘Prediabetes’ (100–125 mg/dL, 5.6–6.9 mmol/L); ‘Healthy’ (50–99 mg/dL); and ‘Low FBG’ (< 50 mg/dL). Based on a modification of the guidelines of the Third Report of the National Cholesterol Education Program (NCEP) [13], study subjects were classified by their measured total cholesterol levels into 5 groups: ‘Extremely high’ (> 360 mg/dl); ‘High’ (240–359 mg/dl); ‘Borderline’ (200–239 mg/dL); ‘Healthy’ (120–199 mg/dl); and ‘Low’ (< 120 mg/dL). Using hemoglobin levels, study subjects were classified into 4 groups: ‘Anemia’ (< 13.0 g/dl in men and < 12.0 g/dl in women); ‘Desirable’ (13–14.9 g/dl in men and 12–13.9 g/dl in women); and ‘High’ (≥ 15 g/dl in men and ≥ 14 g/dl in women). ALT levels were used to determine 3 groups: ‘Low’ (< 20 U/L), ‘Desirable’ (20–39 U/L) and ‘High’ (≥ 40 U/L). Urine occult blood and urine protein detection in the Dipstick test were used as surrogate markers of chronic kidney injury (CKI). A disease score was constructed by the subject’s number of self-reported diseases including heart disease, stroke, diabetes mellitus, liver disease and cancer at baseline. We used 18.5, 23, 25, 27.5, and 30 as BMI (kg/m^2^) cut-off points to enable international comparisons with the WHO [14]. SBP and DBP was used to classify subjects into the group, ‘Healthy’ (SBP < 140 mmHg and DBP < 90 mmHg); ‘Hypertension Stage 1’ (SBP of 140–159 mmHg or DBP 90–99 mmHg); and ‘Hypertension Stage 2’ (SBP of ≥ 160 mmHg or DBP of ≥ 100 mmHg), based on the guidelines of the Seventh Report of the Joint National Committee on Prevention, Detection, Evaluation, and Treatment of High Blood Pressure (JNC 7) [15]. Prehypertension was included in the ‘Healthy’ group. We included the variables age, square of age (age^2^), sex, smoking (pack-years), drinking frequency, disease score from the questionnaire, BMI, FBG, total cholesterol, ALT, hemoglobin, and CKI as surrogate markers to develop the risk predictor. The variables were selected for the general health status based on the current knowledge and how widely they are used for health screening.

Korean medical insurance system evaluates the property and annual household income to provide Medicaid and health insurance services. We used the quintiles of property and annual income as an inequality index.

### Statistical methods

Hazard ratios (HRs) and 95% confidential intervals (95%CIs) for each exposure variable and mortality risk were calculated by multiple Cox proportional hazard models in both traditional and time-variant methods. **Risk score** assessing the probability of each individual’s death was calculated by the product of HRs as follows:

$$\text{risk}\;\text{score}=\prod_{rf}\beta_{rf}\times \left(rf-M_{rf}\right)$$

*β*_*rf*_: Beta coefficient of risk factor

*M*_*rf*_: Mean value of risk factor (mean of each category of risk factor for categorical variables)

Equation 1. Calculation of risk score

The absolute risk of each individual was calculated with risk score and mean survival rate in 10 years.

$$P=1-S{\left(10\right)}^{\text{exp}\left(risk\;score\right)/ME_{rs}}$$

P: absolute risk

S(t): survival rate in t years

ME_rs_: mean of exp (risk score)

Equation 2. Calculation of absolute risk from risk score

In addition to the risk prediction models based on Cox proportional hazard models, other risk prediction models based on boosting and random forest [16, 17] were used. Boosting and random forest are both decision tree based machine learning methods. Boosting makes a decision tree and changes it slightly based on the classification error at each step. Random forest makes several decision trees with randomly selected subgroups.

We calculated the range of 10-year mortality risk and its risk reduction by eliminating a modifiable risk factor at different age groups and sex. The risk reduction was calculated by subtracting each risk factor from the highest risk condition for mortality.

### Validation and sensitivity analysis

The risk prediction model was evaluated by the area under the curve (AUC) of the receiver operating characteristics (ROC) curve for discrimination based on 5-fold cross-validation with bootstrapping and external validation with the Korean Multicenter Cancer Cohort (KMCC), which is a community-based cohort recruited from four regions [18] with study subjects over 40 years of age. To assess the effect of repetitive measurements, we built another model with time-variant Cox regression and compared the two models. The calibration of the model was evaluated with the ratio of the expected and observed number of deaths (expected/observed ratio). For missing value imputation, the two highest rates of missing data for risk factors were 12.6% (smoking pack-years) and 2.9% (exercise frequency). The missing rates of the other risk factors were lower than 1%. The median value for continuous variables and the mode for categorical variables were used to impute missing values for the prediction models using random forest and boosting. Multiple imputation with chained equations (MICE) [19] with 5 imputed datasets and 10 iterations was used to impute the missing values of the KMCC data. The missing values were treated the same as the category with baseline risk. The statistical software packages used were R 3.2 (R Core Team, Vienna, Austria), mice R-package [20], Python 2.7 (Python Software Foundation, Amsterdam, Netherlands) and scikit-learn 0.17 python-package [21].

### Ethical approval

This project was approved by the institutional review board in Seoul National University Hospital (reference number 0909-048-295). There is no consent form because the data were analyzed anonymously.

## Results

The mean age of the 514,320 study subjects at baseline was 53.15 years. The general characteristics of the study subjects and the associations between potential risk factors and risk of all-cause death are presented in Table 1. The differences in estimated HRs either byCox proportional hazard model or by the time-variant Cox regression model were not significant. Age, square of age-40, sex, smoking amount, drinking frequency, past history score, BMI, BP, FBG, total cholesterol, hemoglobin, ALT and surrogate markers of CKI were selected to implement the risk prediction models (S1 Equation). The mean of HRs of the multiple Cox proportional hazard model was used to calculate the risk score. All-cause 10-year survival rate was used to calculate the 10-year mortality risk of the risk score (Table 1).

**Table 1:** Major risk factors for all-cause death among Korean population in the National Health Insurance Service—National Health Screening Cohort (NHIS-HEALS) from 2002 to 2013.

	Person-year	N of Death	HR (95% CI)^1^	HR (95% CI)^2^
Age (years)				
40–44	123,277	2,067	1.00	1.00
45–49	100,135	2,405	1.45 (1.37–1.54)	1.38 (1.32–1.43)
50–54	89,795	3,416	2.42 (2.29–2.56)	2.29 (2.21–2.38)
55–59	57,453	3,344	3.71 (3.51–3.91)	3.56 (3.43–3.69)
60–64	68,622	6,958	7.00 (6.66–7.35)	6.51 (6.29–6.74)
65–69	37,023	6,519	12.77 (12.15–13.41)	12.04 (11.63–12.47)
70–74	27,451	8,135	23.95 (22.82–25.14)	22.17 (21.43–22.93)
75–79	10,564	5,187	46.71 (44.38–49.16)	45.94 (44.38–47.57)
Sex				
Male	2,820,815	25,888	2.32 (2.28–2.36)	2.33 (2.29–2.36)
Female	2,413,414	12,143	1.00	1.00
Cigarette smoking status				
Never	3,380,570	20,993	1.00	1.00
Past	444,752	3,579	1.13 (1.09–1.16)	1.09 (1.06–1.11)
Current	1,191,369	11,891	1.63 (1.60–1.66)	1.64 (1.62–1.67)
Cigarette smoking duration (years)				
0	3,380,570	20,993	1.00	1.00
1–4	97,623	691	1.31 (1.23–1.39)	1.24 (1.18–1.30)
5–9	127,465	633	1.07 (1.00–1.14)	1.06 (1.00–1.11)
10–19	448,218	2,112	1.21 (1.17–1.26)	1.21 (1.17–1.24)
20–29	545,284	3,274	1.36 (1.32–1.40)	1.32 (1.29–1.36)
30+	417,531	8,760	1.66 (1.63–1.70)	1.66 (1.63–1.69)
Cigarette smoking amount (pack/ day)				
< 0.5	295,891	3,984	1.00	1.00
0.5–0.9	632,160	5,699	1.03 (1.00–1.06)	1.01 (0.98–1.03)
1–1.9	246,604	2,044	1.08 (1.03–1.13)	1.05 (1.02–1.09)
2+	16,714	164	1.25 (1.10–1.41)	1.31 (1.19–1.44)
Pack-year of cigarette smoking				
0	3,402,806	21,191	1.00	1.00
1–19	735,597	6,118	1.54 (1.50–1.58)	1.57 (1.54–1.60)
20–39	340,100	4,210	1.70 (1.66–1.75)	1.70 (1.66–1.73)
40+	93,436	1,365	1.77 (1.70–1.85)	1.80 (1.74–1.86)
Alcohol drinking frequency				
Never	2,904,320	21,861	1.25 (1.22–1.29)	1.29 (1.26–1.32)
2–3 / month	793,232	3,842	1.00	1.00
1–2 / week	857,130	5,016	1.13 (1.09–1.17)	1.12 (1.10–1.15)
3–4 / week	361,821	2,901	1.26 (1.21–1.31)	1.29 (1.25–1.33)
Every day or more	219,363	3,666	1.62 (1.56–1.68)	1.68 (1.63–1.72)
Alcohol drinking amount (Soju^3^ bottle/once)				
< 0.5	897,956	6,994	1.00	1.00
1	928,841	6,005	1.09 (1.06–1.12)	1.07 (1.05–1.10)
1.5	254,205	1,269	1.06 (1.01–1.11)	1.02 (0.99–1.06)
2+	127,579	978	1.38 (1.30–1.45)	1.36 (1.31–1.42)
Frequency of regular exercise				
Never	2,903,318	24,896	1.54 (1.48–1.59)	1.60 (1.56–1.64)
1–2 / week	1,217,859	5,781	1.11 (1.07–1.16)	1.14 (1.11–1.18)
3–4 / week	478,885	2,123	1.00	1.00
5–6 / week	134,828	742	1.09 (1.02–1.16)	1.06 (1.01–1.11)
Every day or more	348,321	3,386	1.28 (1.22–1.33)	1.30 (1.26–1.34)
Liver disease at baseline				
No	5,198,932	37,355	1.00	1.00
Yes	35,297	676	2.19 (2.03–2.37)	2.37 (2.26–2.48)
Heart disease at baseline				
No	5,162,341	36,640	1.00	1.00
Yes	71,888	1,391	1.48 (1.4–1.56)	1.50 (1.45–1.55)
Stroke at baseline				
No	5,211,622	37,427	1.00	1.00
Yes	22,607	604	1.82 (1.68–1.97)	1.65 (1.57–1.73)
Diabetes mellitus at baseline				
No	5,021,107	34,319	1.00	1.00
Yes	213,122	3,712	1.63 (1.58–1.69)	1.60 (1.57–1.63)
Cancer at baseline				
No	5,206,708	37,449	1.00	1.00
Yes	27,521	582	1.89 (1.74–2.05)	1.97 (1.88–2.06)
Disease score at baseline				
0	4,333,165	25,894	1.00	1.00
1	781,002	9,532	1.32 (1.29–1.35)	1.29 (1.27–1.31)
2+	120,062	2,605	1.77 (1.70–1.84)	1.69 (1.65–1.73)
Body mass index (kg/m^2^)				
< 18.5	113,470	2,630	2.49 (2.40–2.58)	2.61 (2.54–2.68)
18.5–22.9	1,842,623	15,736	1.38 (1.35–1.41)	1.41 (1.39–1.43)
23.0–24.9	1,432,275	8,865	1.06 (1.03–1.08)	1.07 (1.05–1.09)
25.0–27.4	1,386,237	7,975	1.00	1.00
27.5–29.9	308,873	1,846	1.07 (1.03–1.11)	1.07 (1.04–1.10)
30+	150,751	979	1.29 (1.22–1.36)	1.28 (1.23–1.33)
Systolic and diastolic blood pressures				
Healthy	1,325,851	6,365	1.00	1.00
Hypertension, Stage 1	2,135,193	13,800	1.01 (0.99–1.04)	0.99 (0.97–1.01)
Hypertension, Stage 2	1,771,951	17,854	1.18 (1.16–1.21)	1.18 (1.16–1.21)
Fasting glucose levels (mg/dL)				
< 50	2,046	30	1.74 (1.29–2.35)	1.97 (1.49–2.59)
50–99	3,587,304	21,743	1.00	1.00
100–125	1,238,609	10,076	1.10 (1.08–1.12)	1.10 (1.08–1.12)
126–199	310,863	4,248	1.53 (1.49–1.57)	1.52 (1.49–1.55)
200+	89,221	1,817	2.45 (2.36–2.55)	2.54 (2.46–2.62)
Total cholesterol levels (mg/dL)				
< 120	764	35,806	2.22 (2.1–2.35)	2.20 (2.11–2.30)
120–199	20,508	2,673,312	1.00	1.00
200–239	11,273	1,760,687	0.84 (0.83–0.86)	0.84 (0.82–0.85)
240–359	5,277	748,032	0.91 (0.89–0.93)	0.91 (0.90–0.93)
360+	73	6,964	1.43 (1.20–1.72)	1.36 (1.16–1.59)
Hemoglobin levels (g/dl)^4^				
Anemia	489,115	5,673	1.55 (1.52–1.59)	1.62 (1.59–1.65)
Desirable	3,007,241	21,447	1.00	1.00
High	1,732,465	10,805	0.90 (0.88–0.92)	0.90 (0.89–0.91)
ALT levels (U/L)				
< 20	2,259,898	15,940	1.06 (1.04–1.08)	1.09 (1.08–1.11)
20–39	2,306,927	16,178	1.00	1.00
40+	662,418	5,806	1.50 (1.46–1.53)	1.51 (1.48–1.54)
CKI surrogate marker^5^ (protein/glucose)				
Desirable & Desirable	4,826,054	34,056	1.00	1.00
1+ or 1+	229,160	1,980	1.16 (1.12–1.21)	1.17 (1.14–1.20)
≥ 2+ or ≥ 2+	179,015	1,995	1.45 (1.4–1.50)	1.48 (1.44–1.52)

^1.^ Cox proportional hazard model by baseline exposure to risk factors

^2.^ Time-variant Cox proportional hazard model by repeated exposures to risk factors

^3.^ ‘Soju’ is the most popular alcoholic beverage in Korea. It is clear and colorless and its alcohol content being most common is 20%

^4.^ ‘Anemia’ (< 13.0 g/dl in men and < 12.0 g/dl in women); ‘Desirable’ (13–14.9 g/dl in men and 12–13.9 g/dl in women); and ‘High’ hemoglobin levels (≥ 15 g/dl in men and ≥ 14 g/dl in women)

^5.^ CKI surrogate markers were detected by urine protein/glucose in the Dipstick test

**Fig 1:**
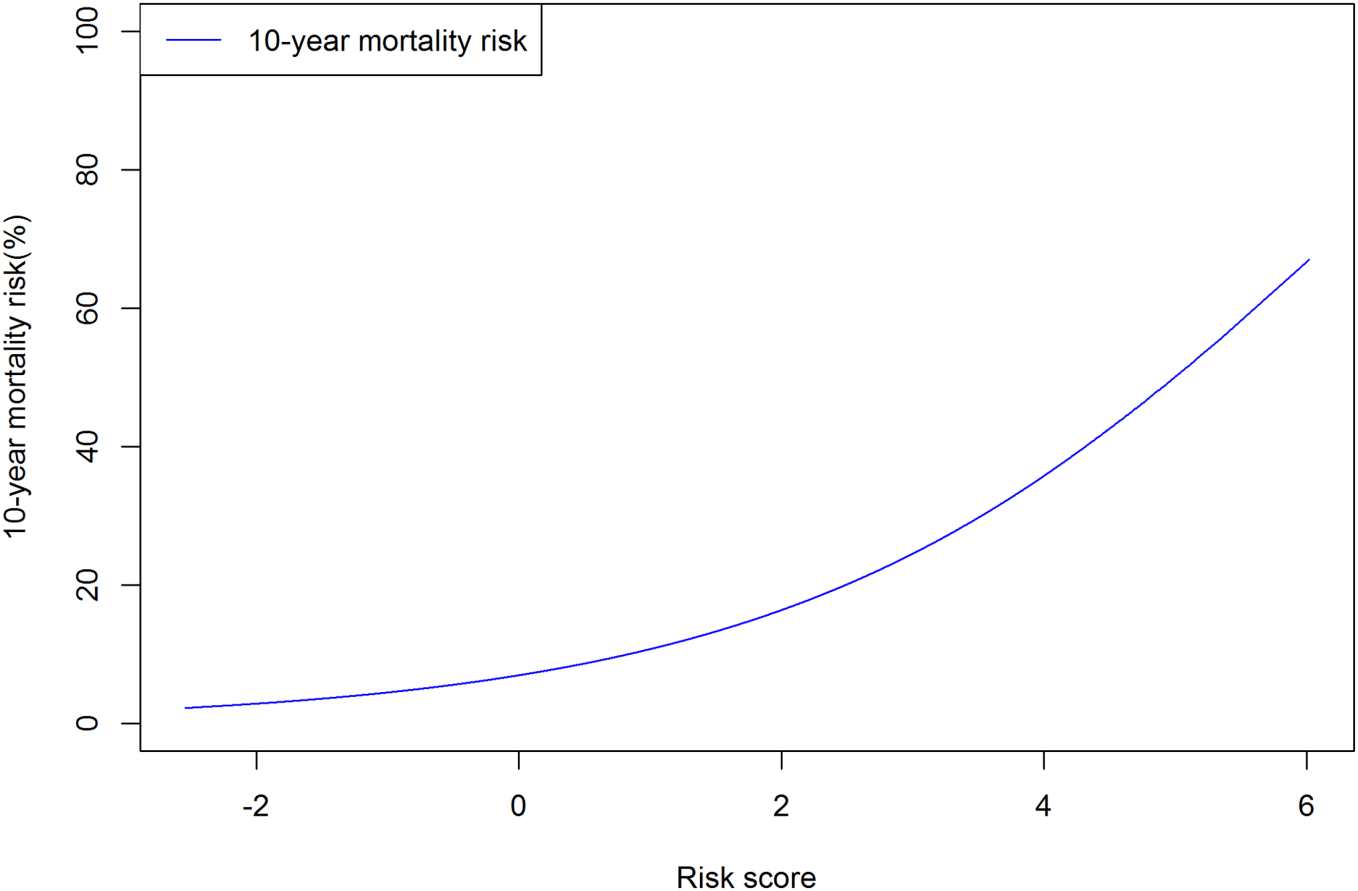
10-year mortality risk in the National Health Insurance Service—National Health Screening Cohort (NHIS-HEALS) from 2002 to 2013.

**Fig 2:**
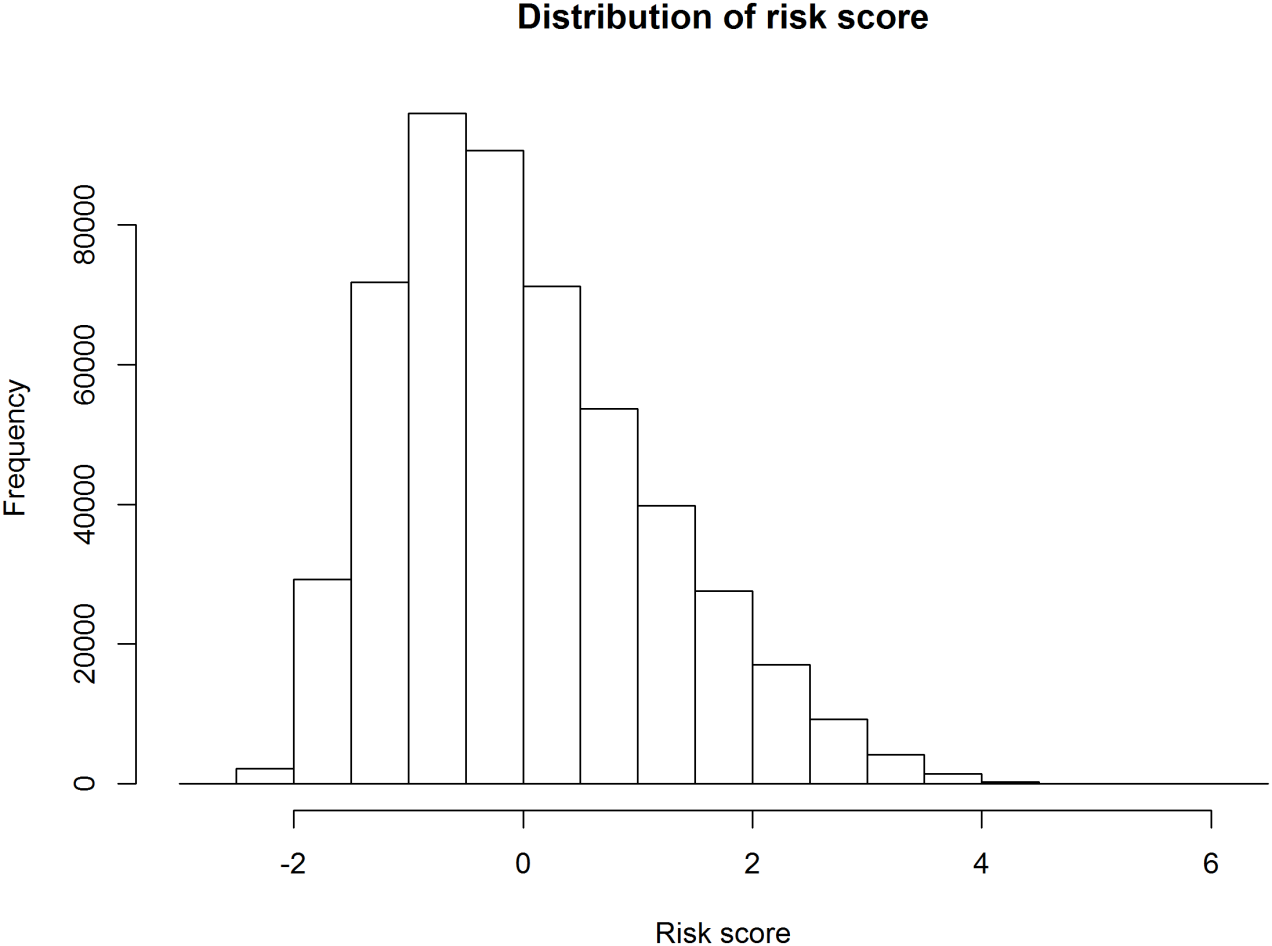
The distribution of the risk score in the National Health Insurance Service—National Health Screening Cohort (NHIS-HEALS) from 2002 to 2013.

Since the average of the risk scores is 0, the proportion of risk scores decrease as the risk scores increase. At higher risk scores (> 1), the proportion of the risk score was low (< 0.2), and at much higher risk scores (> 2), the proportion of the risk score was rare (< 0.1) (Figs 1 and 2). The calibration was good for the total population (expected/observed = 0.894), for women (expected/observed = 0.919) and for men (expected/observed = 0.895).

Table 2 shows the contribution of each modifiable risk factor to the 10-year motarlity risk. Modifying fasting blood glucose had the highest reduction and blood pressure had the lowest reduction on 10-year mortality risk.

**Table 2:** Contribution of major risk factors of all-cause of death risk score in the National Health Insurance Service—National Health Screening Cohort (NHIS-HEALS) from 2002 to 2013.

Sex	Age	Max risk^1^	Min risk^1^	Pack-year ^2^	Drinking frequency ^2^	Frequency of regular exercise ^2^	Disease score at baseline ^2^	BMI ^2^^,^ ^3^	BP ^2^^,^ ^4^	FBG ^2^^,^ ^5^	Total cholesterol levels ^2^	Hemoglobin levels ^2^	ALT levels ^2^	CKI surrogate markers ^2^
**Male**	40	50.7	1.4	0.6	0.1	0.1	0.7	1.1	0.1	1.2	1	0.5	0.5	0.4
**Female**	40	40.1	1	0.5	0.1	0.1	0.5	0.8	0.1	0.9	0.8	0.4	0.4	0.3
**Male**	45	65.3	2	1	0.3	0.3	1.1	1.7	0.3	1.9	1.6	0.8	0.8	0.6
**Female**	45	53.6	1.5	0.7	0.2	0.2	0.8	1.2	0.1	1.4	1.1	0.5	0.6	0.4
**Male**	50	80.6	3.1	1.6	0.4	0.4	1.7	2.5	0.4	2.9	2.4	1.2	1.2	1
**Female**	50	69.6	2.3	1.1	0.3	0.2	1.2	1.8	0.2	2.1	1.7	0.8	0.9	0.7
**Male**	55	92.7	5	2.4	0.6	0.5	2.6	3.8	0.5	4.5	3.7	1.7	1.9	1.4
**Female**	55	85	3.6	1.8	0.5	0.4	2	2.9	0.4	3.4	2.8	1.3	1.4	1.1
**Male**	60	98.7	8	3.8	1.1	0.9	4.2	6.1	0.8	7.1	5.9	2.8	3	2.3
**Female**	60	95.6	5.9	2.8	0.8	0.7	3.1	4.6	0.6	5.3	4.4	2.1	2.2	1.7
**Male**	65	99.9	13.3	6	1.6	1.4	6.5	9.5	1.3	11	9.2	4.4	4.7	3.6
**Female**	65	99.5	9.8	4.6	1.3	1.1	5	7.3	1	8.5	7.1	3.4	3.6	2.8
**Male**	70	100	22.1	9.2	2.6	2.2	10	14.4	2	16.5	13.9	6.9	7.3	5.6
**Female**	70	100	16.6	7.3	2	1.7	7.9	11.5	1.6	13.2	11.1	5.4	5.8	4.4

^1^ Maximum and minimum 10-year mortality risk (%)

^2^ 10-year mortality risk reduction caused by each modifiable risk factor (%)

^3^ Body mass index

^4^ Blood pressure

^5^ Fasting blood glucose

The c-statistics using Cox proportional hazard and time-variant Cox proportional hazard models were 0.832 and 0.841, respectively. The c-statistics of the machine learning-based random forest and boosting models were 0.893 and 0.712, respectively. The discrimination ability of the prediction model decreased with age (c-statistics = 0.82 for subjects aged 40–49 years; 0.78 for those aged 50–59; 0.72 for those aged 60–69; and 0.69 for those aged 70+). The c-statistics for the external validation using a community-based cohort, KMCC, was 0.814 (Table 3).

**Table 3:** Results from cross validation with bootstrapping and external validation of risk prediction models to estimate 10-year mortality risk by the combination of major risk factors of all-cause of death in the National Health Insurance Service—National Health Screening Cohort (NHIS-HEALS) from 2002 to 2013.

	C-statistics (95% CI)
**Cross validation**	
**Cox PHM**^1^	0.832 (0.831–0.834)
**Time-variant Cox PHM**^1^	0.841 (0.840–0.842)
**Random forest**	0.893 (0.795–0.992)
**Boosting**	0.712 (0.412–1.000)
	
**External validation**	
**Korea Multicenter Cancer Cohort**	0.814 (0.805–0.823)

^1^ proportional hazard

Subjects with a high income level had relatively low 3-year and 10-year all-cause mortality risks and comprised a lower proportion of the high-risk group defined by various risk score cutoffs (S1–S3 Tables).

## Discussion

In this large, population-based national cohort study, we evaluated the associations between health information and measurements that can be obtained from routine health check-ups and 10-year all-cause mortality and developed a mortality risk predictor. Although the prediction models were developed with health information and measurements based on self-reported data including smoking, drinking, exercise, and medical history, anthropometric measurements, and blood and urine laboratory test results, the all-cause mortality risk predictor showed excellent discrimination with cross and external validation.

The Korea National Health and Nutrition Examination Survey (KNHANES) [22] and several other health screening programs [23–25] previously used these health information and measurements for the health check-up program. Although general health check-ups cannot reduce all-cause mortality [1, 2], repeated check-ups can be used to improve the surrogate markers of mortality [2]. Prior results support our finding that the health information and measurements obtained in general health check-up can be used to estimate a future individual death risk.

The discriminatory ability of our prediction model was higher than the mortality predicted by general self-rated health only (c-statistics = 0.74) or by the Vulnerable Elders Survey (VES-13) for the elderly (c-statistics = 0.78) [26, 27]. The discriminatory ability of the random forest model relative to that of the traditional or time-variant Cox regression models was higher for predicting mortality (c-statistics = 0.89) despite the wide confidence intervals of the c-statistics, whereas the discriminatory ability of the boosting model was rather low (c-statistics = 0.71). The major reason for this difference is the difference between the two machine-learning models in classification algorithms; boosting is usually known to work better in shallow trees (5–15 leaves) and with data from many weak learners (a classifier that is only slightly correlated with the true classification) [28]. Therefore, a lower discriminatory ability for boosting is consistent with its innate algorithm.

Prior studies have reported U- or J-shaped associations of all-cause mortality with BMI (underweight, normal weight, and obesity) [29]; cholesterol (low, high, total and LDL-cholesterol) [30, 31]; FBG (low FBG or impaired glucose levels, diabetes levels) [32, 33]; and hemoglobin (low or very high) [34], while for physical activity and cigarette smoking, an increase in all-cause mortality with higher levels of exposure to these variables has been reported [35, 36]. Undernutrition may be a highly predictive factor of short-term mortality, especially in the elderly [37], and low cholesterol, FBG, hemoglobin, and BMI may be phenotypes of undernutrition. In our data, those with lower levels of the four surrogate markers had a lower SES, smoked more cigarettes, drank alcohol more frequently, exercised less frequently and had a past history of 0.75 diseases on average. By contrast, those with higher levels of FBG, BMI, cholesterol and hemoglobin had a higher SES, exercised more frequently and had a past history of 0.93 diseases on average, and they were more vulnerable to long-term death than short-term death.

A previous study developed a risk prediction score using many self-reported health indicators and some blood assays [10], while we developed a risk predictor based on health information and measurements composed of self-reported data, blood and urine assays, and anthropometric measurements that are commonly used in periodic general health check-ups.

Both models can help the adult population seeking health information by providing proper health information regarding health determinants to estimate their future individual risk of death and by improving self-awareness of proper interventions to maintain good health despite increases in anxiety and overutilization. Additionally, physicians would be able to provide suggestions for modifying lifestyles using the mortality risk predictor as quantitative evidence. Moreover, a reduction in mortality risk at the population level could be expected from targeted interventions for high-risk individuals or groups based on individual mortality risk.

Our study has several limitations. First, the risk predictor was calibrated for individuals who were enrolled in the national health screening program and were 40–70 years old. Additional calibration is required before it can be generalized. Second, we developed a risk predictor for all-cause mortality, and thus additional risk predictors for cause-specific mortality may be developed in the future. Since the risk predictor was developed based on the general health examinees who are relatively healthy, the predictability of the developed risk predictor in variant health conditions has to be investigated before it can be generalized. Lastly, we did not include the control group of subjects who would not attend check-ups in the analyses. For this reason, it was difficult to evaluate how periodic check-up itself influence individuals’ mortality risk. Therefore, we also need to interpret the data considering potential bias of socio-economic status or employment status.

In summary, we developed a 10-year all-cause mortality risk predictor based on data from a national health screening program conducted with the middle-aged to elderly population in Korea. We developed a risk prediction model based on common measures obtained by questionnaires, basic physical examinations and blood tests. The risk predictor developed in this study showed better discriminatory ability than previous predictors. Further trials are needed including studies determining the availability of health information and measurements in younger populations, site-specific mortality risk and risk of disease incidence, and validation of the predictor in other populations should continue to be researched.

## Supporting information

S1 Table 10-year and 3-year death probability by income and prior diseases among Korean population in the National Health Insurance Service—National Health Screening Cohort (NHIS-HEALS) from 2002 to 2013. (DOCX)

S2 Table 10-year and 3-year death probability by income and prior diseases among Korean population in the National Health Insurance Service—National Health Screening Cohort (NHIS-HEALS) from 2002 to 2013. (DOCX)

S3 Table 3- and 10-year death probability by cut-point with good performance among Korean population in the National Health Insurance Service—National Health Screening Cohort (NHIS-HEALS) from 2002 to 2013, by income group. (DOCX)

S4 Table Multivariate odds of high risk (> cut-point 0.5) 10-year death risk by income, prior diseases among Ko.rean population in the National Health Insurance Service—National Health Screening Cohort (NHIS-HEALS) from 2002 to 2013, by income and prior diseases. (DOCX)

S1 Equation Calculation of risk score and 10-year mortality risk based on risk score. (DOCX)
