# Supplementary material for: Predictors of all-cause mortality among 514,866 participants from the Korean National Health Screening Cohort
Source: PLoS One. 2017 Sep 28;12(9):e0185458. doi: 10.1371/journal.pone.0185458 (PMC5619780; doi:10.1371/journal.pone.0185458)
Supplement: S1 Table — (DOCX) [file pone.0185458.s001.docx]

**S1 Table. 10-year and 3-year death probability by income and prior diseases among Korean population in the National Health Insurance Service - National Health Screening Cohort (NHIS-HEALS) from 2002 to 2013**

| Income level^1^ | 3-year Death | 10-year Death |
| --- | --- | --- |
|  | % (95% CI) | % (95% CI) |
| Total |  |  |
| Q1 | 2.18 (2.08-2.29) | 8.97 (8.76-9.19) |
| Q2 | 1.98 (1.88-2.08) | 8.06 (7.86-8.25) |
| Q3 | 1.54 (1.46-1.62) | 6.69 (6.53-6.86) |
| Q4 | 1.29 (1.23-1.35) | 5.64 (5.52-5.76) |
| Q5 | 1.08 (1.01-1.15) | 5.06 (4.92-5.2) |
|  |  |  |
| Men |  |  |
| Q1 | 3.63 (3.42-3.83) | 14.22 (13.83-14.6) |
| Q2 | 2.82 (2.65-2.98) | 10.97 (10.66-11.28) |
| Q3 | 2.00 (1.88-2.12) | 8.32 (8.08-8.55) |
| Q4 | 1.60 (1.51-1.69) | 6.60 (6.43-6.77) |
| Q5 | 1.27 (1.18-1.37) | 5.52 (5.33-5.71) |
|  |  |  |
| Women |  |  |
| Q1 | 1.01 (0.91-1.11) | 4.70 (4.49-4.91) |
| Q2 | 1.12 (1.01-1.23) | 5.05 (4.83-5.27) |
| Q3 | 0.92 (0.83-1.02) | 4.52 (4.31-4.72) |
| Q4 | 0.85 (0.78-0.93) | 4.30 (4.13-4.47) |
| Q5 | 0.77 (0.68-0.86) | 4.31 (4.1-4.52) |

**^1^**Income levels were categorized into quintiles based on NHIS house income database
