# Supplementary material for: Predictors of all-cause mortality among 514,866 participants from the Korean National Health Screening Cohort
Source: PLoS One. 2017 Sep 28;12(9):e0185458. doi: 10.1371/journal.pone.0185458 (PMC5619780; doi:10.1371/journal.pone.0185458)
Supplement: S2 Table — (DOCX) [file pone.0185458.s002.docx]

**S2 Table. 10-year and 3-year death probability by income and prior diseases among Korean population in the National Health Insurance Service - National Health Screening Cohort (NHIS-HEALS) from 2002 to 2013**

| Prior diseases | 3-year Death | 10-year Death |
| --- | --- | --- |
|  | % (95% CI) | % (95% CI) |
| Total |  |  |
| 0 | 1.27 (1.24-1.31) | 5.55 (5.48-5.62) |
| 1 | 2.59 (2.48-2.69) | 10.91 (10.71-11.11) |
| 2 | 4.35 (4.03-4.67) | 17.9 (17.29-18.50) |
| 3 | 6.42 (5.34-7.5) | 23.42 (21.53-25.26) |
| Cancer | 6.90 (5.98-7.82) | 19.31 (17.86-20.73) |
|  |  |  |
| Men |  |  |
| 0 | 1.72 (1.66-1.77) | 7.11 (7.00-7.21) |
| 1 | 3.47 (3.32-3.63) | 13.67 (13.37-13.97) |
| 2 | 5.52 (5.01-6.02) | 21.77 (20.85-22.68) |
| 3 | 8.55 (6.8-10.27) | 28.97 (26.10-31.74) |
| Cancer | 11.43 (9.67-13.15) | 30.95 (28.38-33.43) |
|  |  |  |
| Women |  |  |
| 0 | 0.74 (0.70-0.78) | 3.67 (3.58-3.75) |
| 1 | 1.60 (1.48-1.71) | 7.84 (7.59-8.09) |
| 2 | 3.17 (2.78-3.56) | 14.00 (13.22-14.77) |
| 3 | 4.27 (3.00-5.53) | 17.80 (15.38-20.16) |
| Cancer | 3.32 (2.45-4.19) | 10.09 (8.62-11.55) |
