# Supplementary material for: Predictors of all-cause mortality among 514,866 participants from the Korean National Health Screening Cohort
Source: PLoS One. 2017 Sep 28;12(9):e0185458. doi: 10.1371/journal.pone.0185458 (PMC5619780; doi:10.1371/journal.pone.0185458)
Supplement: S3 Table — (DOCX) [file pone.0185458.s003.docx]

**S3 Table. 3- and 10-year death probability by cut-point with good performance among Korean population in the National Health Insurance Service - National Health Screening Cohort (NHIS-HEALS) from 2002 to 2013, by income group**

| Cut-point | 3-year Death | 10-year Death | Cut-point | 3-year Death | 10-year Death |
| --- | --- | --- | --- | --- | --- |
|  | % (95% CI) | % (95% CI) |  | % (95% CI) | % (95% CI) |
| Total | 1.63 (1.60-1.67) | 6.99 (6.92-7.06) |  |  |  |
| -0.5 |  |  | 0.5 |  |  |
| < -0.5 | 0.28 (0.26-0.31) | 1.20 (1.15-1.25) | < 0.5 | 0.51 (0.49-0.54) | 2.22 (2.17-2.27) |
| ≥ -0.5 | 2.48 (2.43-2.54) | 10.65 (10.54-10.76) | ≥ 0.5 | 4.26 (4.16-4.36) | 18.22 (18.03-18.41) |
|  |  |  |  |  |  |
| 0 |  |  | 1 |  |  |
| < 0 | 0.39 (0.36-0.41) | 1.65 (1.61-1.7) | < 1 | 0.67 (0.65-0.70) | 2.93 (2.88-2.98) |
| ≥ 0 | 3.24 (3.16-3.31) | 13.88 (13.74-14.03) | ≥ 1 | 5.61 (5.47-5.75) | 23.88 (23.61-24.14) |
