# Supplementary material for: Predictors of all-cause mortality among 514,866 participants from the Korean National Health Screening Cohort
Source: PLoS One. 2017 Sep 28;12(9):e0185458. doi: 10.1371/journal.pone.0185458 (PMC5619780; doi:10.1371/journal.pone.0185458)
Supplement: S4 Table — (DOCX) [file pone.0185458.s004.docx]

**S4 Table. Multivariate odds of high risk (> cut-point 0.5) 10-year death risk by income, prior diseases among Korean population in the National Health Insurance Service - National Health Screening Cohort (NHIS-HEALS) from 2002 to 2013, by income and prior diseases**

|  | Total | Men | | Women |
| --- | --- | --- | --- | --- |
|  | High risk | High risk | | High risk |
|  | OR (95% CI) | OR (95% CI) | | OR (95% CI) |
| Income |  |  |  |  |
| Medicaid |  |  | |  |
| Q1 | 1.61 (1.57-1.64) | 3.11 (3.02-3.2) | | 0.89 (0.86-0.92) |
| Q2 | 1.42 (1.39-1.45) | 2.08 (2.02-2.13) | | 0.93 (0.9-0.96) |
| Q3 | 1.16 (1.14-1.19) | 1.39 (1.35-1.42) | | 0.9 (0.87-0.93) |
| Q4 | 0.99 (0.97-1.01) | 1.00 (0.98-1.03) | | 1 (0.97-1.03) |
| Q5 | 1.00 | 1.00 | | 1.00 |
|  |  |  | |  |
| Prior diseases |  |  |  |  |
| 0 | 1.00 | 1.00 | | 1.00 |
| 1 | 2.86 (2.81-2.9) | 2.93 (2.88-2.99) | | 3.08 (3.01-3.15) |
| 2 | 7.99 (7.71-8.28) | 9.04 (8.56-9.56) | | 8.72 (8.31-9.15) |
| 3 | 14.99 (13.37-16.86) | 17.7 (14.66-21.58) | | 16.38 (14.15-19.03) |
| Cancer | 3.51 (3.26-3.78) | 7.06 (6.22-8.03) | | 2.64 (2.38-2.92) |
|  |  |  | |  |
| Body mass index (Kg/m2) |  |  | |  |
| < 18.5 | 3.19 (3.07-3.31) | 3.69 (3.49-3.91) | | 2.98 (2.82-3.15) |
| 18.5 - 22.9 | 1.00 | 1.00 | | 1.00 |
| 23 - 24.9 | 0.68 (0.67-0.69) | 0.5 (0.49-0.51) | | 0.99 (0.96-1.01) |
| 25 - 27.4 | 0.74 (0.72-0.76) | 0.5 (0.48-0.52) | | 1.25 (1.2-1.3) |
| 27.5 - 29.9 | 0.89 (0.86-0.92) | 0.62 (0.59-0.66) | | 1.49 (1.42-1.56) |
| 30+ | 0.68 (0.67-0.69) | 0.5 (0.49-0.51) | | 0.99 (0.96-1.01) |
|  |  |  | |  |
| Fasting plasma glucose levels (mg/dL) |  |  | |  |
| < 50 | 2.96 (2.25-3.9) | 3.16 (2.19-4.57) | | 2.64 (1.69-4.05) |
| 50 - 99 | 1.00 | 1.00 | | 1.00 |
| 100 - 125 | 1.65 (1.63-1.68) | 1.43 (1.4-1.45) | | 1.89 (1.84-1.93) |
| 126 - 199.9 | 3.96 (3.87-4.06) | 3.25 (3.16-3.35) | | 4.84 (4.65-5.03) |
| ≥ 200 | 7.93 (7.58-8.31) | 7.32 (6.89-7.79) | | 8.22 (7.66-8.83) |
