# Supplementary material for: Predictors of all-cause mortality among 514,866 participants from the Korean National Health Screening Cohort
Source: PLoS One. 2017 Sep 28;12(9):e0185458. doi: 10.1371/journal.pone.0185458 (PMC5619780; doi:10.1371/journal.pone.0185458)
Supplement: S1 Equation — (DOCX) [file pone.0185458.s005.docx]

S1 Equation. Calculation of risk score and 10-year mortality risk based on risk score.

Risk score = (Age – 53.153) X 0.0779 + ((Age-40)^2^ – 266.7256) X 0.0006276

+ ([Sex=Male]– 0.5422) X 0.7005

+ ([Pack-year>0 & Pack-year<20] – 0.1610) X 0.4423 + ([Pack-year>=20 & Pack-year<40] – 0.0759) X 0.5147 + ([Pack-year>=40] – 0.0211) X 0.51

+ ([Drinking frequency=never] – 0.5673) X 0.0617+ ([Drinking frequency=1-2/week] – 0.1652) X 0.1900+ ([Drinking frequency=3-4/week] – 0.0706) X 0.0653+([Drinking frequency=everyday] – 0.0446) X 0.1163

+ ([Exercise frequency=never] – 0.5748) X 0.3329+ ([Exercise frequency=1-2/week] – 0.2364) X 0.3154+ ([Exercise frequency=5-6/week] – 0.0263) X 0.0971+ ([Exercise frequency=everyday] – 0.0694) X 0.0896

+ ([Past history score=1] – 0.1525) X 0.2476+ ([Past history score>=2] – 0.0245) X 0.4870

+ ([BMI<18.5] – 0.0233) X 0.7460 + ([BMI>=18.5 & BMI<23] – 0.3541) X 0.2964 + ([BMI>=23 & BMI<25] – 0.2723) X 0.0707 + ([BMI>=28 & BMI<30] – 0.0586) X 0.0186 + ([BMI>=30) – 0.0287] X 0.1414

+ ([SBP >= 140 or DBP >= 90] – 0.4064) X 0.0522 + ([SBP >= 160 or DBP >= 100] – 0.3425) X 0.1860

+ ([Fasting blood glucose<50] – 0.0004) X 0.4963 + ([Fasting blood glucose>=100 & Fasting blood glucose<126] – 0.2377) X 0.1020 + ([Fasting blood glucose>=126 & Fasting blood glucose<200] – 0.0611) X 0.3337 + ([Fasting blood glucose>=200] – 0.0180) X 0.7278

+ ([Total cholesterol<120] – 0.0074) X 0.5409+ ([Total cholesterol>=200 & Total cholesterol<240] – 0.3350) X -0.1076 + ([Total cholesterol>=240 & Total cholesterol<360] – 0.1427) X -0.0518 + ([Total cholesterol>=360] – 0.0013) X 0.1730

+ ([Hemoglobin <13 for male, <12 for female] – 0.0957) X 0.3124 + ([Hemoglobin >=15 for male, >=14 for female] – 0.3285) X -0.0957

+ ([ALT<20] – 0.4322) X -0.0225 + ([ALT>=40] – 0.1269) X 0.3710

+ ([CKD surrogate marker = 1+ or 1+] – 0.0443) X 0.1012+ ([CKD surrogate marker = 2+ or higher] – 0.0350) X 0.2757

10-year mortality risk = 1 – 0.9300902^exp(Risk score)/2.21^
